# Inequities in mental health and mental healthcare between international immigrants and locals in Chile: a narrative review

**DOI:** 10.1186/s12939-020-01312-2

**Published:** 2020-11-04

**Authors:** Alice Blukacz, Báltica Cabieses, Niina Markkula

**Affiliations:** 1 Instituto de Ciencias e Innovación en Medicina, Facultad de Medicina, Clínica Alemana, Universidad del Desarrollo, Avenida Las Condes 12461, Las Condes, Región Metropolitana Chile; 2 Department of Psychiatry, Faculty of Medicine, University of Helsinki and Helsinki University Hospital, P.O.Box 22, 00014 Helsinki, Finland

## Abstract

Mental health in a context of international migration is a particularly pressing issue, as migration is recognised as a social determinant of physical and mental health. As Chile is increasingly becoming a receiving country of South-South migration, immigrants face mental health inequities, with regards to outcomes and access to care.

In order to identify and synthetize mental healthcare inequities faced by international migrants with regards to locals in Chile, a narrative review of the literature on national mental healthcare policies in Chile and a narrative review of the literature on migrants’ mental healthcare in Chile were conducted, with a focus on describing mental health outcomes, policy environment and persisting gaps and barriers for both topics. The existing literature on mental healthcare in Chile, both for the general population and for international migrants, following the social determinant of health framework and categorised in terms of i) Inequities in mental health outcomes; ii) Description of the mental health policy environment and iii) Identification of the main barriers to access mental healthcare.

Despite incremental policy efforts to improve the reach of mental healthcare in Chile, persisting inequities are identified for both locals and international migrants: lack of funding and low prioritisation, exacerbation of social vulnerability in the context of a mixed health insurance system, and inadequacy of mental healthcare services. International migrants may experience specific layers of vulnerability linked to migration as a social determinant of health, nested in a system that exacerbates social vulnerability.

Based on the findings, the article discusses how mental health is a privilege for migrant populations as well as locals experiencing layers of social vulnerability in the Chilean context. International migrants’ access to comprehensive and culturally relevant mental healthcare in Chile and other countries is an urgent need in order to contribute to reducing social vulnerability and fostering mechanisms of social inclusion.

International migration, social determinants of mental health, mental health inequities, social vulnerability, review.

## Background

Global challenges in prevention and treatment of mental health problems remain only partially addressed. According to the World Health Organization (WHO), between 35 and 50% of people with severe mental disorders in high income countries receive no treatment, rising to between 76 and 85% in low and middle-income countries [1]. In the Americas, mental and substance use disorders account for 10.5% of the global burden of disease [2], and according to the same study, 71.2% of people with a mental disorder are not treated.

Chile is a high-income Latin American country and a leading economy in the region. However, 20.5% of its population experiences multidimensional poverty and 4.4% of the population in rural areas experiences extreme poverty [3]. With regards to health provision, it is important to note that the Chilean healthcare system is mixed, with a public health insurance system, *Fondo Nacional de Salud* (FONASA), which provides healthcare for 70% of the population and a private health insurance system, *Instituciones de Salud Previsional* (ISAPRE) accounting for 25% of the population [4]. The rest of the population is covered by the Armed Forces and Police insurance system, which has its own infrastructure and suppliers, and is funded through general taxation [5]. Additionally, there is a growing market for private complementary and supplementary health insurance [6].

Eligibility for public coverage is non-conditional on health status or income, however private health insurance companies can reject applicants if their financial contribution does not match their estimated health risk [7]. In that sense, 1.6% of people experiencing poverty report being covered by the private health insurance system while 15.6% of non-poor people report private health coverage. Although the public system, in theory, should guarantee a universal coverage, effective access to healthcare may depend on the ability to pay for private care, due to inefficiencies in the underfunded and ill-equipped public system [8, 9].

Patients covered by FONASA are primarily female, elderly and low-to-middle income earners, with a greater prevalence of risk factors and a lower health status, suggesting adverse selection. Despite having greater health needs, FONASA beneficiaries have lower service utilisation than ISAPRE beneficiaries [10]. This mixed system, set up during the military dictatorship in the 1980s, has been increasingly questioned in the past few years [11, 12] despite the 2004 Law N°19.966 on Explicit Health Guarantees (*Ley N°19.966 de Garantías Explícitas de Salud,* thereafter GES), approved in the context of the Universal Access and Explicit Guarantees reform (*Acceso Universal con Garantías Explícitas,* AUGE thereafter). The GES law and AUGE reform arguably represented an effort towards a more inclusive healthcare coverage in an exclusive and segmented system, by guaranteeing that both the public and private insurance schemes provide basic treatment for 80 prioritised illnesses [13–15]. Considering the inequities described in the overall healthcare system, this paper focuses specifically on the mental health outcomes of the general population, the evolution of the mental healthcare system at national level, and persisting gaps in the model, in order to situate the challenges faced by international migrants both with regards to their mental health outcomes, needs, and barriers to accessing care in the national context.

Chile has been increasingly receiving immigrants mainly from Latin America and the Caribbean region in the last decade, with the estimated number of foreign residents reaching 1,492,522 (8% of the population) at the end of 2019 [16]. The number of approved temporary and permanent residence permits increased from 19,588 in 2000, to 328,111 in 2019 [16]. The majority of migrants are from Spanish-speaking countries such as Venezuela (30.5%), Peru (15.8%) and Colombia (10.8%), with the exception of immigrants from Haiti (12.5%) who usually speak Creole and French. 71% of the immigrant population lives in the Metropolitan Region of Santiago. Temporary visas can be granted on different basis, among which family relations with a Chilean citizen or permanent foreign resident, employment including a specific visa for highly-skilled professionals, investors, and citizens of the Southern Common Market (MERCOSUR) [17]. Although the majority of visa applications fall under those categories, a growing number of asylum applications has been registered since 2015, when 623 applications were submitted, a number reaching 5727 in 2018, before falling again in 2019. The three main countries of origin for asylum applicants are Colombia (43.7%), Cuba (29,7%) and Venezuela (21,5%) [18].

With regards to sex, women are only very slightly overrepresented (51.4%). Importantly, international migrants tend to be younger than the local population, as 75.7% of them are between 15 and 44 years-old, while only 40.8% of the Chilean-born population is represented in this age group [19]. In terms of educational level, international migrants over 18 have, on average, completed more years of education than the Chilean-born – 13.2 and 11.1 years respectively [19]. The five main sectors employing international migrants are wholesale and retail, hotel and restaurant industry, real estate, domestic work and construction [19]. Although not all international immigrants in Chile are necessarily socioeconomically vulnerable, as a group, they tend to experience more aspects of social vulnerability, and to a higher degree, than the local population. The rate of multidimensional poverty among the foreign-born was 24.6%, or four percentage points above people born in Chile.

The increasing number of immigrants in Chile brings new challenges, among which immigration management [20], public health [21] and social inclusion [22–24], for a country that had, until the last couple of decades, been one of emigration rather than immigration. In this context of a growing and increasingly diverse inflow of international migrants to Chile, access to healthcare has gained attention in the last few years. Decree N°67 (*Decreto Supremo N°67*), which came into force in June 2016, gives migrants access to the basic coverage under FONASA regardless of their migration status, and in the same conditions as Chileans with no source of income [4]. Additionally, the International Migrant Health Policy (*Política de Salud de Migrantes Internacionales*) was implemented in 2018, bringing attention to the particular needs of migrant populations with regards to access, quality and relevance of healthcare. Further examination of the existing policies for international migrants’ access to mental healthcare is carried out in our review.

Despite efforts by the Chilean government to develop and implement policies aimed at improving access to healthcare for the migrant population, the data available suggests that international migrants in Chile do not have access to healthcare to the same level of the Chilean-born population. The most recent study conducted with data from the 2017 National Socioeconomic Characterization Survey (*Caracterización Socioeconómica Nacional*, CASEN) found that 16.3% of international immigrants in Chile were not beneficiaries of any of the health insurance modalities available, compared to 2.3% of the Chilean population, and that 80% of the insured immigrants was in the public system, while 18% was in the private system. However, immigrants reported a lower perceived need to access healthcare, but also presented less odds of obtaining an appointment, less coverage and lower satisfaction of the service received with regards to need in the short and long-term. Finally, the same study found that immigrants had 2.7 higher odds of not receiving coverage for an AUGE/GES disease, a rate that is 3.3 times higher than for Chileans and shows horizontal inequity between the local population and international migrants [4]. Access to mental healthcare is an emerging priority, with little attention given at policy level. Migrants in Chile report limited access to mental healthcare, although there is no evidence that their need is lesser than that of the Chilean born population [25].

In the context of health promotion and policymaking for vulnerable groups such as international migrants, the notion of “layers of -social and socioeconomic- vulnerability” that has taken prominence in the field of bioethics in recent years [26] in order to understand the particular forms of social vulnerability experienced by different migrant subgroups is relevant. Identifying the layers of vulnerability experienced by international migrants with regards to mental health, requires first identifying mental health and mental healthcare inequities in Chile. Our review looks at the existing literature on mental health in Chile in terms of inequities in outcomes and access to care, first at national level for the general population, before turning to international immigrants. The general policy environment for mental healthcare in Chile is described, as is the policy environment for international migrants’ access to mental healthcare. This review contributes to the literature on migrants’ mental health and social inclusion, highlighting the multidimensional aspects of access to mental healthcare in a context of human mobility. It informs policy makers and public health practitioners interested in social inequities in health on the urgency of improving the access and cultural relevance of mental healthcare for migrant populations.

## Methods

### Data search and selection

Carrying out a narrative review was selected in favour of carrying out a systematic review, in order to achieve a broad and inclusive overview of the policy environment for international migrants’ access to mental healthcare in Chile and of the existing evidence on the barriers they face, based on a diverse body of literature, including original studies as well as policy documents and protocols at national level.

First, a narrative review of the existing literature on mental health in Chile was conducted. A search was carried out on PubMed and Google Scholar in both English and Spanish and the key words used in the search were: “mental health system AND Chile”, “mental healthcare AND Chile”, “mental health policy AND Chile”. The search was iterative, whereby relevant articles included in the bibliography of the journals initially selected were included. Thirty-two journal articles published between 2003 and 2018 focusing on the Chilean mental healthcare system were taken into account. We allowed a broad selection of types of articles as long as they clearly included mental health in Chile, including original research, both qualitative and quantitative, descriptive case studies and published expert opinions from relevant public health actors in Chile. Additionally, one national mental health plan, one clinical protocol and a report, all from the Ministry of Health of Chile were included.

Second, a narrative review of the literature on migrants’ access to physical and mental healthcare in Chile was conducted, in order to describe the existing policies and identify the barriers faced by international migrants for accessing mental healthcare specifically. The choice was made to include literature on the barriers faced by international migrants to access healthcare in general, and not exclusively mental healthcare, in order to situate the barriers identified in the specific literature in a broader context of barriers to access to healthcare. A search was thus carried out on PubMed and Google Scholar in both English and Spanish, and the key words used in the search were: “migrant AND healthcare AND Chile”, “migrant AND access AND healthcare AND Chile”, “migrant AND mental health AND Chile”. As in the first review, the search was iterative and a hand search of relevant references from selected papers was included. In total, 18 journal articles were taken into account, published between 2011 and 2020, as well as one health plan and a study report, both from the Ministry of Health of Chile. As in the first review, we included a diverse selection of types of articles as long as they clearly included the health or mental health of international migrants in Chile, including original research, both qualitative and quantitative, descriptive case studies and published expert opinions from relevant public health actors in Chile.

### Data analysis

Social determinants of health can be contextual, with policies at national level impacting public and individual health. They can also stem from living and working conditions as well as community influence, individual lifestyle choices and living and working characteristics [4, 27, 28]. With regards to mental health, under a biopsychosocial approach, Compton & Shim argue that the “role that nongenetic social and environmental factors play in bringing about poor mental health and in causing and worsening mental illnesses (page 419)”, including “the role of social justice, political will and power, policy action, resource distribution, and program development and implementation in addressing these factors (page 419)” must be taken into account [29].

Migration is recognised as a crosscutting social determinant of health [30–32]. During all stages of the process of migrating, the health of migrants might improve or worsen due to a range of factors, such as immigration, social and public health policies, living and working conditions, social exclusion, discrimination and stigma, as well as educational attainment, socioeconomic class, legal status and cultural barriers [4, 27, 28]. Migration is also a social determinant of mental health, depending on the conditions of pre-migration, migration and post-migration processes [33–35] and interactions with broader social determinants of health [36].

In that sense, we analysed the existing literature on mental health in Chile, both for the general population and for international migrants, following the social determinant of health framework. For each corpus of literature the results are presented as follows:

- Inequities in mental health outcomes;
- Description of the mental health policy environment;
- Identification of the main barriers to access mental healthcare.

## Results

### Inequities in mental health outcomes in Chile

According to the Burden of Disease and Attributable Burden Study (*Estudio de Carga de Enfermedad y Carga Atribuible*) conducted in 2008 by the Ministry of Health of Chile, 23.2% of the Years of Life Lost because of disability or death were due to neuro-psychiatric conditions [37]. In 2016, the OECD attributed 25.5 deaths per 100,00 inhabitants to mental and behavioural disorders in Chile [38] and for the same year, the suicide rate was 10.7 per 100,000 inhabitants [39]. However, the burden of disease and prevalence of disorders described in the data presented in the previous paragraph may not be shared equally among the population, and inequities in mental health outcomes can originate from different, sometimes interacting layers of social vulnerability. This section examines the main social determinants of mental health and the dimensions of social vulnerability in Chile identified in the existing literature.

#### Dimensions of social vulnerability and inequities of mental health outcomes

With regards to demographic characteristics, it seems that in Chile, gender, and particularly being a woman is a determinant of mental health status, as women are two to three times more at risk of developing suicidal behaviour [40]. Furthermore, women tend to use the psychiatric sick leave provision of FONASA more than men, and their leave periods are 12% longer on average [41]. With regards to age, the prevalence of psychiatric disorders is high among children and teenagers [42].

In line with existing global evidence [43], socioeconomic background and status seem to act as a social determinant of mental health in Chile, as socioeconomic status was found to be significantly associated with a low prevalence of anxiety disorders [42], and a higher prevalence of common mental disorders was found among the most socially disadvantaged groups, in particular after a recent income drop [44]. More specifically, with regards to education, there is a strong inverse association between educational attainment and the prevalence of common mental disorders and there is an inverse relationship between levels of education and suicidal behaviour, and between income and suicidal behaviour [40].

Another aspect of social vulnerability, beyond socioeconomic background and status is social capital. In the Chilean context of a highly unequal society with a rigid social and occupational class structure [45], social capital is positively associated with job market attainment, and there is a relationship between socioeconomic background, social capital, and status attainment through direct and indirect associations [46]. More particularly, social capital, can be used as an “umbrella term” encompassing social cohesion, support, integration and participation and has been positioned among the social determinants of physical and mental health [47]. Under that broad definition, the relevance of social capital for mental health can be brought back to Durkheim’s study of suicide and the notion of social suicide rate, whereby suicide rates can be explained in relation to social integration [48]. In general terms, regarding the effect of social capital on mental health status and the effect of mental health status on social capital, Sartorius highlights the effect that social capital might have on low self-esteem and self-confidence as a consequence of mental disorders [49]. A systematic review of primary research found that individual social capital, usually measured through individuals’ participation in social relationships and perceptions of the quality of these relationships, tended to be negatively assessed by respondents suffering depression and anxiety disorders. Conversely, at ecological level, there is no clear evidence of an inverse relationship [50]. While few studies regarding the general population in Chile have been conducted, there is evidence of strong associations between social capital indicators and depression [51], and of a positive relationship between social capital and mental health status in low-income urban communities, suggesting that social capital has indeed an impact on mental health [52].

With regards to occupation and mental health, retail workers in Chile are the most likely to ask for sick leave for psychiatric reasons, while construction workers are the least likely to do so [41]. In the domestic work sector, poor mental health among female domestic workers is linked to self-esteem and the perception they have, and are given, that their work is undervalued and shameful [53]. This becomes relevant especially in relation with the evidence presented on socioeconomic background and social capital.

The existing literature in Chile provides us with a context on the inequities of mental health outcomes where variables such as gender, age, socioeconomic background, social capital and more specifically occupation create what we can call “layers of social vulnerability”. Having examined the inequities in mental health outcomes, we can now turn to describing the evolution of the Chilean Mental Healthcare Model.

### Evolution of the Chilean mental healthcare model

#### National mental health programmes and plans: towards a community approach to mental health in primary healthcare

In line with the Caracas Declaration of 1990, WHO recommendations, as well as standards of human rights, the Chilean mental healthcare system is largely decentralised, based on community mental health teams [54, 55] and integrated in primary healthcare [56]. Efforts to address the mental health of the general population in Chile started in the 1960s with the launch of the National Mental Health Programme (*Programa Nacional de Salud Mental*) and subsequent initiatives to implement programmes of community-based psychiatry [57], which were interrupted by the military coup of 1973 [58].

After democracy was re-established in 1990, the government took measures aimed at reinforcing the healthcare system and put emphasis on the social and psychological dimensions of health, with the creation of the Mental Health Unit in the Ministry of Health and a network of mental health professionals [59]. Additionally, the need to address mental health more widely and systematically came into light through surveys aimed at primary healthcare users undertaken in the early 1990s [56]. This culminated in the approval, in 1993, of the First National Mental Health and Psychiatry Plan (*Primer Plan Nacional de Salud Mental y Psiquatría*), which established the basis for the integration of mental healthcare in primary healthcare [59].

The 2000 Mental Health and Psychiatry Plan (*Plan de Salud Mental y Psiquiatría*) was built upon the experience and approach of the previous Plan in terms of the integration of mental healthcare in primary healthcare [60] and decentralisation through the strengthening of networks of healthcare centres providing mental healthcare [54]. This focus on providing mental healthcare through primary healthcare allowed to expand its reach [55] and increase the detection, diagnosis and treatment of mental disorders [54]. Additionally, the inclusion of mental healthcare in primary healthcare was taken a step further with the National Depression Treatment Programme of 2001 [61, 62]. In that sense, mental health policy was aligned with the main recommendations of the *WHO World Health Report 2001 – Mental Health: New Understanding, New Hope* [63], which stressed the importance of primary healthcare and the community approach to mental healthcare [64].

The current National Mental Health Plan, the *Plan Nacional de Salud Mental 2017–2025* launched in 2017, introduces seven strategic lines of action aimed at improving alignment with standards of human rights, defining guidelines and strategies to improve access to mental healthcare according to the needs of the population; developing a plan for sustainable and efficient funding for the implementation of programmes to promote mental health; improving systems for quality management, monitoring and investigation; increasing the amount of mental healthcare workers and improving their work conditions; promoting the participation of civil society in policy-making; and promoting an intersectoral approach to mental health. The Plan emphasises the community-based approach to mental healthcare as well as adherence to standards of human rights [37].

#### The inclusion of mental healthcare in broader health policy

In the last 15 years, broader healthcare policies and policy instruments have also explicitly included mental healthcare, such as the *Chile Crece Contigo* programme in 2008, which includes the promotion of mental health from pregnancy until the child is 9 years old [32]. With regards to general health, the National Health Strategy 2011–2020 (*Estrategia Nacional de Salud para el Cumplimiento de los Objetivos Sanitarios de la Década 2011–2020)* also includes, among 50 health goals, four goals related to mental health [65].

Although there is no specific law for the promotion of mental health, several existing laws address issues linked to mental health [66]. In terms of access to mental healthcare, the 2004 Law N°19.966 on AUGE and GES stands out, as it guarantees that both the public and private insurance schemes must ensure basic treatment for 80 prioritised illnesses, among which schizophrenia, depression, alcohol and drug abuse and bipolar disorder [56, 65–67]. Access to treatment for depression increased after the introduction of AUGE/GES, especially among women and socioeconomically disadvantaged groups [68].

### Persisting gaps: funding, social determinants of mental health and inadequacy

#### Funding and prioritisation

In the context of the segmented healthcare system, lack of financial resources allocated to mental healthcare is a recurring topic among the existing literature on mental healthcare in Chile [63, 67, 69–71]. Particularly with regards to the public system, the WHO-AIMS 2014 report on the Chilean Mental Healthcare System reported that the percentage of the healthcare budget allocated to mental healthcare was 2.16% in 2012, whereas the average in upper-middle income countries was 2.38% and 5.10% in high income countries [66].

On the other hand, mental healthcare specialist Alberto Minoletti and colleagues show the progress made in terms of funding for mental healthcare. The allocation of specific funds for mental health in primary healthcare since 2000 allowed for programmes to be rolled out all over the country and to reach parity of mental health funding mechanisms with regards to physical health in primary healthcare since 2015. However, they admit that funding remains insufficient [60]. Moreover, it is important to note that the issue is not limited to the public sector, as most plans in the private health insurance system offer a much more modest coverage for mental healthcare than physical healthcare [65].

Beyond lack of financial resources, the AUGE/GES has been criticised for the inequities it might unintentionally create, as the prioritisation of certain diseases represents the “rationing” of the right to health [14]. Only four mental disorders are currently included among the 80 diseases prioritised, and only half of the disorders that had been prioritised in the 2000 Mental Health Plan is covered by AUGE/GES [65]. In that sense, mental health does not appear to be a priority and equal access to treatment is limited.

Finally, the Mental Health Plan for 2017–2025 recognises the urgent need for a Mental Health Law in Chile in order to bring together and harmonise the existing norms, promoting the social inclusion of people with mental diseases, community-based mental healthcare, and intersectoral measures to address the social determinants of mental health, in line with human rights standards and principles [37]. According to the Plan, provisions should also be made regarding the funding of mental healthcare in order to fill the existing gaps in terms of promotion, prevention, diagnosis, treatment and recovery, eliminate discrimination based on affiliation to the public or private sector and ensure that mental disorders be given the same priority as other disorders.

Scarcity of funding is not a standalone factor however and the existing literature also points to the intersection between structural and individual factors that determine access to mental healthcare in the context of a segmented healthcare system.

#### Social vulnerability and segmentation: social determinants of access to mental healthcare

Following the social determinants of health framework, policies and contextual factors at national level can exacerbate social vulnerability at individual level, when for instance, eligibility for a more comprehensive healthcare insurance is directly linked to wealth and health status, as is the case in the Chilean segmented system, which leads most of its critics to focus on the inequities created by segmentation.

In that sense, with regards to inequities in mental healthcare, data from the 2010 National Health Survey (*Encuesta Nacional de Salud*, ENS) shows that 21% of patients suffering depression were receiving antidepressant treatment at the time of the survey, and 48.9% had received treatment at some point in their lifetime [72], suggesting a gap between needs and treatment. However, this gap may not be experienced equally by everyone, with differences in consultation rates between patients covered by public FONASA and private ISAPRE, which are in turn greater among those with the most severe symptom or a high degree of disability [7], as well as geographical inequities, where the “level of accessibility, quality of care and the community orientation depends primarily on where a person lives (page 765)” [55].

Considering the segmented healthcare system, and the fact that those who might not have access to private ISAPRE due to low income as well as higher health needs, have, to borrow Araya et al’s words, “little choice other than to remain in the underfunded public sector, where they are least likely to receive professional help (page 113)” [7]. Finally, other cultural factors, such as stigma towards mental illness [73], which might have an effect on seeking and accessing treatment especially within lower-income groups [74] or the idea that mental health might be seen as a private issue which should be dealt with by the individual and their family, rather than becoming a policy priority [65].

Access to mental healthcare is thus inequitable and conditional to several, sometimes intersecting social determinants. Furthermore, access does not necessarily guarantee the adequacy of the service and treatment received.

#### Inadequacy of mental health services

Mental healthcare in Chile is largely integrated in primary healthcare, which arguably improved access, considering that FONASA beneficiaries receive free mental health attention in primary healthcare centres [70]. There is, however a lack of strategy for monitoring quality and promoting improvement within primary healthcare, meaning that some patients might receive inadequate treatment with regards to both their needs and the existing scientific evidence [56, 60]. There is, additionally, a lack of efficient monitoring mechanisms to follow-up clinical outcomes and adherence to treatment [60].

Although broadly promoted at policy level, the community approach to mental healthcare is limited in practice, as there is a lack of agency and participation of the communities involved and a subsequent lack of utilisation of local and cultural resources and knowledge to improve mental healthcare [56], as well as limited approach to wider social interests in the democratisation of mental healthcare [75]. Furthermore, psychologists in primary healthcare centres find themselves focusing on their daily work and immediate individual assistance, with little space left for a more holistic approach around prevention and protection including family members, communities and risk groups [76].

In terms of the adequacy of therapy, it is important to note that although the AUGE reform and GES law have been praised for improving access to treatment, they include pre-established protocols for the treatment of the prioritised diseases. In that sense, although a patient might be diagnosed with one of the four prioritised mental health diseases, they might require a different treatment than that specified in the protocols [15]. For instance, the treatment for schizophrenia defined in the 2005 guide established by the Ministry of Health was mainly centred on antipsychotic drugs while psychosocial interventions were administered complementarily [77], and although the guide was updated in 2017, clinician’s adherence was higher for the former than for the latter in the application of the 2005 guide [72]. In that same line, a highly biomedical and individual approach to mental healthcare remains, and the role of social determinants of health in mental health status and outcomes is underplayed, despite efforts to integrate it into community-level healthcare [78].

Despite incremental policy efforts to increase funding for mental healthcare in the public system and reduce barriers to access to care for a number of mental health disorders through the implementation of AUGE/GES and the expansion of the network of mental health professionals in primary healthcare, the Chilean mental healthcare model displays persisting gaps for equitable access due to the exacerbation of social vulnerability in the context of a segmented health insurance system and the inadequacy of certain aspects of the delivery of mental healthcare. In this context, social determinants of health have an effect on both mental health status and access to mental healthcare.

### Inequities in mental health outcomes for international migrants in Chile

Although the pre-departure and transit phases are crucial in determining the mental health outcomes of migrants, the data on the socioeconomic background of international migrants prior to their departure to Chile is scarce and so is the work on conditions of transit. In that sense, our review focuses on processes of acculturation in the receiving country and their link with mental health. Following the concept of social layers of vulnerabilities, international migrants may experience the factors leading to inequities in mental health outcomes described in the dedicated section for the general population in Chile. The factors described here are specific to international migrants and are considered in addition to the other social determinants of mental health in Chile.

#### Acculturation and mental health outcomes

The concept of acculturation is defined by Berry as the cultural changes resulting from the encounter and processes of adaptation of individuals who have developed in a given cultural context in a new context as a result of migration, as a result of which individuals might experience psychological acculturation, or psychological changes [79]. Acculturation strategies are integration (retaining the culture of origin while adopting the host culture), assimilation (withdrawing from the culture of origin and adopting the host culture), separation (retaining the culture of origin and rejecting the host culture) and marginalisation strategies (rejecting both cultures) [80, 81].

These strategies result from two attitudes towards acculturation: striving for cultural maintenance or the extent to which contact and participation are considered, which in turn can be determined by the attitude of the “receiving” group towards the other, one of which can be discrimination [79]. In turn, acculturation strategies can have an impact on mental health status, in the form of acculturative stress as defined by Berry et al. as a reduction in psychological health status at individual level as a result of processes of contact with the “dominant group”, or society of settlement [82, 83].

A systematic review of papers on common mental disorders among immigrants around the world was conducted, and its results indicated that the prevalence of these disorders increases with perceived discrimination and low levels of acculturation, among other factors [84]. The link between discrimination and the mental health of immigrants and ethnic minorities has been defined around variables such as time since arrival [85], ethnicity [86], and the experience of discrimination and humiliation [87, 88].

#### Discrimination, acculturation strategies and mental health in Chile

In the case of Chile, several studies highlight this link, and international migrants may experience both social vulnerability, reporting discrimination, and psychosocial vulnerability, with symptoms of anxiety and depression [89]. One of them, focused on Peruvian migrants in Santiago, found that perceived individual discrimination was a stressor and determinant of poor mental health, especially for women [90]. There is, furthermore, a positive relationship between discrimination and anxiety and depression, as found among Peruvian and Colombian migrants in Arica, Antofagasta and Santiago, which can however be mitigated by self-esteem [91].

In relation with acculturation, integration can lead to better psychological wellbeing, and assimilation can lead to better overall wellbeing, as found among South American migrants in Antofagasta y Calama in Northern Chile, albeit with differences among nationalities [92]. With regards, specifically, to strategies of acculturation and mental health, a study found that Peruvian migrants, who usually employ strategies of assimilation or bicultural integration, tend to display more symptoms of common mental disorders than Colombians, who employ strategies of separation. This is explained by the difference in terms of the possible negative experience of discrimination and rejection that migrants who choose to assimilate or integrate might face when in contact with the mainstream local culture [93]. A similar study found that sources of acculturation stress include discrimination and perceived rejection, as well as other factors of social vulnerability [94].

No comparable study exists to date about Venezuelan and Haitian immigrants in Chile, although they now constitute two of the main groups of international migrants in the country following recent waves of migration. However, Rojas Pedemonte et al. describe strategies of avoidance and negation of racism and exclusion by Haitian migrants in Santiago [95]. Further research on possible impacts on mental health could be conducted for these specific groups.

### Evolution of the policies for migrants’ access to mental healthcare in Chile

#### International migrants’ right to health care in Chile

All foreigners with a valid residence permit have access to healthcare in Chile [96], however their affiliation to either the public or the private system depends on health status and wealth, as for the Chilean-born population. The first direct measure taken towards the inclusion of migrants into healthcare regardless of legal status was the Oficio Circular N°1.179 of 2003 granting access to undocumented pregnant women through a specific visa [97]. A more recent breakthrough is the Decree N°67 (*Decreto N°67*) that came into force in June 2016, giving irregular migrants equal access to FONASA with regards to nationals if they have no source of income [4].

As immigration peaked in Chile, with 438,223 residence permits granted in 2018 [16] and migrants’ health as a matter of public health gained more attention, the Ministry of Health launched the International Migrant Health Policy (*Política de Salud de Migrantes Internacionales*) the same year. The Policy recognises migration as a social determinant of mental health in terms of the discrimination migrants suffer in Chile, the negative narratives around migration, as well as the loss of family relationships and difficult living and working conditions. In that sense, it specifies the inclusion of migrants as a group whose members might experience different forms of social and socioeconomic vulnerability into strategies for the promotion of mental health in one of its strategic guidelines on the inclusion of international migrants’ health into public health programmes and interventions [98].

#### Addressing the mental health of international migrants

The International Migrant Health Policy recommends developing cross-cultural capacities to improve mental healthcare for this particular group. Moreover, the current National Mental Health Plan (*Plan Nacional de Salud Mental 2017–2025*) recognises that mental healthcare must consider the inclusion of a cross-cultural approach more specifically for Native communities (*pueblos originarios*), while highlighting the inclusion of cross-cultural and language facilitators in primary healthcare [37].

Migrants’ access to mental health care in Chile is an emerging topic and the existing literature is relatively limited. However, recent research has described access to mental healthcare and identified the barriers that migrant populations are facing. This strand of literature shows that migrants’ access to mental healthcare is affected both by the systemic gaps in the Chilean mental healthcare system described previously and by the specific barriers to healthcare that they face as foreigners.

### Barriers to international migrants’ access to mental health care in Chile

#### Funding, prioritisation and discrimination: specific challenges for international migrants

The review of the literature on the overall mental health care model in Chile showed that lack of funding and prioritization of mental health was an issue especially for the public sector, although coverage gaps for mental health in the private health insurance system meant that access is limited, overall. For international migrants, there are additional systemic barriers in the overall public health care system, such as lack of information regarding the number of international migrants and their healthcare needs of international migrants, leading to a lack of resources allocated to already underfunded public healthcare centres to address the needs of that specific population group [99]. In that sense, it is possible that both funding gaps intersect to hinder to international migrants’ access to mental healthcare.

With regards specifically to mental health, Astorga-Pinto et al. provide an overview of the main barriers to access faced by immigrants based on a secondary analysis of qualitative data collected between 2014 and 2016 among migrants and healthcare professionals in eight socially and economically vulnerable areas. One of the barriers identified at systemic level and consistently with the literature on mental healthcare in Chile, is the limited availability of mental health professionals as a consequence of low funding, and low prioritisation of mental healthcare [25]. This barrier was also identified in a study conducted with migrant teenagers in 2019 in the Independencia, Recoleta and Santiago boroughs of the Metropolitan Region of Santiago, where the reason given by the interviewees for not seeking mental healthcare or not adhering to treatment, was that it was not adequate quantitatively, in terms of the length and frequency of the therapy sessions received [100].

Low prioritisation of mental healthcare specifically for international migrants may also be reflected in situations of discrimination and complex perceptions of who is entitled to care in a context of scarcity, as international migrants reported perceived discrimination and fear of being turned down when asking for mental healthcare when needed, based on the perception that they cannot claim entitlement to healthcare in Chile, which is in turn linked to a feeling of not fully belonging [25]. Likewise, perceived and real discrimination may be exacerbated depending on country of origin or ethnicity, following patterns of structural racism and anti-immigrant narratives, whereby international immigrants may be hierarchised according to underlying beliefs and perceptions regarding their country of origin, language and ethnicity, leading to health inequities [101]. Further research should however be undertaken with regards to instances of discrimination in mental healthcare, with a special focus on international migrants from different countries and ethnic backgrounds.

#### Social vulnerability and segmentation: social determinants of access to mental healthcare for international migrants

Regarding the social determinants of health identified in the previous section and their relation to the Chilean segmented healthcare system, female immigrants are more likely to be beneficiaries of the public system than men, 68,4% and 61,7% respectively [21]. Although there has been a decrease in the rate of immigrants reporting having no health insurance, immigrants with disabilities are more likely to report no healthcare or insurance than Chileans, indicating horizontal inequity (less attention and resources despite similar needs). Sociodemographic factors also play a role in the type of provision, with female immigrants living in rural areas being the most likely to be covered only by the most basic provision of the public system [102].

Similarly, socioeconomically deprived immigrants are usually located in areas of the Northern regions of Tarapacá and Antofagasta and the Metropolitan Region of Santiago, where the local population also experiences socioeconomic deprivation [103], which, following the factors of inequities to access to mental healthcare in Chile identified previously, means they may not have de facto access to sufficient and adequate mental healthcare. The first epidemiological study on migrants’ mental health status and access to care in Chile was conducted by Rojas et al. between 2007 and 2008 and focused on adults, youth and children in the Independencia borough in Santiago found that 36% of parents perceiving that their child suffered from a mental disorder reported cost, perception that the issue would resolve itself and lack of knowledge around the Chilean healthcare system as reasons not to seek help. Moreover, when the study was conducted, 30% of the migrants interviewed reported not having access to health insurance, citing this as another important barrier [104]. Considering that that the study was conducted before Decree N°67 came into force, it could be expected that administrative barriers would have been reduced.

There is, however, no evidence that the Decree eliminated all administrative barriers for access to healthcare. In that sense, migrants and health professionals alike report administrative barriers linked to irregular status, despite Decree N°67, as both groups lack knowledge and awareness regarding irregular migrants’ right to access healthcare [105]. Another aspect of social vulnerability linked to migratory status is the fear of deportation reported by international migrants as a reason not to seek mental healthcare [25].

#### Language and culture as barriers to access and acceptability

Our review on the subsisting gaps in the overall Chilean Mental Healthcare Model identified lack of adequacy and flexibility in established mental health treatments, meaning that patients might receive inadequate treatment with regards to their needs, as well as a limited engagement with the community around patients, in terms of participation, prevention and protection. In that overall context, international migrants experience barriers to access to mental healthcare, which are sometimes linked to their perception of relevance and acceptability of the care provided in terms of culture and language.

The issue of intercultural understanding with regards to mental health may begin even before mental healthcare is delivered. A study carried out in 2016 focused on a programme aimed at promoting access of the migrant population to primary healthcare (*Programa de Atención Inicial a Migrantes*), found that very few of the programme participants were referred to mental healthcare units despite the fact that they had been assessed for suicidal behaviour and symptoms of depression. A possible explanation put forward by the authors is that there are different conceptions of mental illness across different cultures, whereby international migrants might not find receiving attention necessary [21]. Similarly, but with regards to healthcare in general, there are cultural barriers regarding perceptions and beliefs around health as reported by health professionals [99]. Further research should be carried out with regards to cultural perceptions around mental health in the context of international migration in Chile, and its impact on access to mental healthcare and cross-cultural care.

Language barriers have been identified as a main obstacle to healthcare in general, [99] and are undoubtedly relevant for mental healthcare. Although the majority of international migrants in Chile are native Spanish-speakers, Chilean Spanish is characterised by distinctive colloquial phrases which are used across social classes and usually regardless of the setting, including, in this case during medical consultations, and may not be understood by other native Spanish-speakers [106].

In terms of the adequacy of the care provided, negative perceptions on healthcare, both physical and mental, as reported by international migrants in Chile focus on the lack of cultural pertinence as well as the failure to address their perceived needs. They cited the lack of cross-cultural understanding between themselves and healthcare professionals, leading to misunderstandings and frustration from both sides, as well as the lack of knowledge and training to deliver mental healthcare relevant to the causes of the mental health disorders they were facing, especially factors linked to forced migration [25, 105]. In addition to providing culturally relevant care and improving mental healthcare professionals’ cross-cultural knowledge and approach, mental healthcare should be focused on addressing these specific factors [107].

Although the evidence on barriers to migrants’ access specifically to mental healthcare in Chile is scarce, and there are limitations, especially with regards to instances of discrimination and perceptions of mental health across cultures, in this section we have situated the barriers to migrants’ access to mental healthcare in the overall model of access to mental healthcare and context of migrants’ access to healthcare. The gaps in the overall mental healthcare model described in the existing literature were categorised into funding and prioritisation, social vulnerability and segmentation, and inadequacy of mental health services, and the barriers faced by international migrants to access mental healthcare were categorised the same way in order to identify intersections and highlight the ways in which international migrants may experience specific layers of vulnerability linked to migration as a social determinant of health, nested in a system that exacerbates social vulnerability.

## Discussion

The commitment to improve the mental health and well-being of the world population is reflected in the inclusion of mental health as part of the Sustainable Development Goals (SDGs) specifically within target 3.4 of SDG 3, “Good Health and Wellbeing”, which requests that countries: “By 2030, reduce by one third premature mortality from noncommunicable diseases through prevention and treatment and promote mental health and well-being” [108]. Mental health inequities, both in terms of outcomes and access, are of concern for both local populations experiencing social vulnerability and international migrants whose experience of social vulnerability may intersect with that of the local population, while being exacerbated by different factors linked to migration as a social determinant of health. Advancing towards the SDGs calls for an urgent need to address mental health inequities with specific attention to vulnerable groups, among which international migrants.

Our review of the existing literature on mental healthcare in Chile shows that it is limited in quantity and adequacy overall, and even more so for international migrants, who experience specific needs and barriers to access. Although the mental healthcare model emphasises decentralised, community-level care and guarantees regarding the treatment of a number of mental diseases, it represents a low proportion of public spending, it is given lower coverage by private insurance than physical healthcare and it is parsimoniously and inadequately delivered through AUGE/GES. Finally, the segmentation of the health insurance system exacerbates both horizontal and vertical inequities.

### Privilege, social vulnerability and mental health

Privilege is defined as “a right or immunity granted as a peculiar benefit, advantage, or favour”, [109] denoting, for those who do not possess it, a disadvantage. The notion of privilege is often brought up in terms of race, “white privilege” or gender, “male privilege”. Privilege is multidimensional and can stem from, and be conceived in terms of, race, class, gender, age, religion, sexual orientation, economic status, social status, health status as well as, in the context of human mobility, migratory status and country of origin. Moreover, instances of privilege mirror layers of vulnerability. Specifically, in Chile, the notion of privilege is conceptualised by Araujo as a rationale, which is not explicitly legitimated but prevalent in the structure and dynamics of relationships, linked to rights violations and to a weak applicability of the principle of equality [110]. In that respect the notion of privilege when analysing mental health inequities is relevant, both in comparison with the Chilean-born population and across different migrant subgroups and our review points to mental healthcare in Chile as a privilege both for the vulnerable local population and international migrants. The latter may experience some of the instances of social vulnerability experienced by the former that determine, on the one hand poorer mental health outcomes and increased needs, and on the other hand, less access to mental healthcare, which in turn may not be qualitatively adapted to improve outcomes. In that sense, the mental healthcare challenges of these two population groups may intersect. However, international migrants also experience specific layers of social vulnerability with regards to their mental health: acculturation stress as a factor of common mental disorders, as well as barriers linked to perceptions of entitlement to mental healthcare in a context of limited funding and prioritisation of mental healthcare, social determinants of access to mental healthcare for international migrants in the context of a segmented healthcare system, and finally lack of cross-cultural and linguistic relevance.

### Acculturation, social inclusion and mental health

The implications of acculturative stress, assimilation strategies and discrimination for the mental health status of international migrants in Chile, as well as the barriers experienced for access to mental healthcare, suggest that there is an urgent need to, on the one hand, improve realized access, and the other hand, improve the adequacy of mental health services. Mental health equity in a context of human mobility is also crucial to the sociocultural inclusion of international migrants and vice-versa. Cross-cultural understanding and relevance in mental healthcare could contribute to improving both the perception of rejection international migrants in Chile report as a barrier to accessing healthcare, as well as the acculturation stress and related symptoms of mental health disorders experienced. One of the main barriers reported was lack of cultural relevance, in a general context of inflexible and inadequate approach to mental healthcare under the AUGE/GES regime. In that sense, and considering that assimilation, or the withdrawal from the culture of origin to adopt the host culture, is used as an acculturation strategy with negative repercussions on mental health status, encouraging cross-cultural mental healthcare for international migrants in Chile could improve both access to mental healthcare and mental health status. Delivering culturally relevant mental healthcare implies building cultural bridges, for instance through building mental healthcare professionals’ intercultural competence, developing culturally adapted communication strategies and encouraging a cross-cultural therapeutic relationship with patients [111]. Adequate mental healthcare could be a way to address and ease acculturative stress and allow for bi-cultural integration through culturally sensitive mental health services.

Furthermore, as the literature on mental health for the general population in Chile showed, social capital may be one aspect of the layers of social vulnerability experienced by the more vulnerable groups of the local population, with effects on their mental health status. With regards specifically to international migrants, dynamics around social capital can also play a role in mechanisms of sociocultural exclusion and poor mental health. Work on acculturation, social capital and mental health has been carried out in the United States with regards to Mexican Americans and Latinos, and greater cognitive social capital at individual level among Mexican American women was associated with fewer depression and anxiety symptoms and that social capital increased with acculturation [112], while social capital as social support mechanisms for recently arrived Latinos reduced acculturation-related stress [113].

When discussing the effect of social capital on mental health status and vice versa, Sartorius points to the link between the growth of social capital and mental healthcare, whereby ‘the successful management of a mental illness’, understood as the prioritisation of access to mental healthcare at policy level and the support given by closer communities and extended networks, as well as recovery, depend directly on social capital [49]. Conversely, according to McKenzie et al., attention needs to be put on processes of vertical integration for social integration [114]. Vertical integration is a “linking” relationship, whereby groups interact with different levels of power and resources, for instance a migrant community interacting with representatives of local governance [115]. Despite the persisting gaps in the mental healthcare system in Chile, one of the strengths identified is its emphasis on community-level care and integration into primary care [54–56]. In addition to comprehensive and culturally relevant mental healthcare for international, vertical integration through community integration and participation in decision-making around mental health is also crucial in mechanisms of social inclusion.

However, promoting social inclusion processes is not a task to be solely taken up by the healthcare sector and that requires participation of different sectors of society and levels of governance. From the perspective of social determinants of health, migration can affect the health status of the migrant in different ways [28], and social and community aspects such as separation from family and the weakening or loss of support networks, can be factors affecting migrant health. Efforts towards community organization and empowerment through active inclusion and participation in bottom-up decision-making could reinforce vertical integration, build intercultural bridges, and enhance social inclusion.

## Conclusions

Our review first examined mental health inequities in terms of outcomes among the Chilean population and persisting gaps in the overall mental healthcare model in Chile. The gaps identified in the overall mental healthcare model point to lack of funding and prioritisation of mental health at policy level, the exacerbation of social vulnerability in the context of a segmented healthcare system and inadequacy of mental healthcare services. In this context, international migrants may experience, depending on their particular background, characteristics and situation, layers of social vulnerabilities that intersect with those of the local population, in addition to social vulnerabilities stemming from being an international migrant in Chile. This has a direct repercussion on access to mental healthcare, as well as outcomes. Access to mental healthcare is a “privilege” insofar as the barriers are multidimensional and most impervious for those who need it most. It is, however, an urgent need in order to foster equity in health and opportunities, as well as social inclusion.

Although this review highlights an emerging challenge in the context of South-South migration, it is not unique to Chile, as societies become increasingly diverse as a result of human mobility, globally, regionally and even nationally. Addressing the mental healthcare of non-nationals and ethnic minorities, and adapting it to foster cross-cultural health exchanges, can reduce discrimination in mental healthcare, and promote mental health as a tool for successful social inclusion is valuable in contexts of cultural diversity and layered social vulnerability. However, addressing the barriers for migrants’ access to mental healthcare linked to social vulnerability in a context of a segmented healthcare system requires systemic changes both in the mental healthcare model and the health insurance system as a whole, which has yet to take place.

## Data Availability

All data generated or analysed during this study are included in this published article.
